# Cocaine-induced midline destructive lesion with extensive cutaneous involvement^؆^

**DOI:** 10.1016/j.abd.2025.501277

**Published:** 2026-01-23

**Authors:** Laís Guerra Guedes, Márcio Martins Lobo Jardim, Felipe Marinho Rocha de Macedo, Isabella Cavalcanti Gomes de Sá, Renata Guerra Galvão Santos, Daniela Mayumi Takano

**Affiliations:** Recife Center for Dermatological Studies, Santa Casa de Misericórdia, Recife, PE, Brazil

Dear Editor,

It is described in the literature that cocaine use is responsible for the development of an entity called cocaine-induced midline destructive lesion (CIMDL).^1^ This report describes the case of a 34-year-old female patient, a chronic cocaine user, who developed CIMDL with extensive cutaneous involvement over a period of eight months, presenting with symptoms such as rhinorrhea and local pruritus before the appearance of a well-defined, painful necrotic ulcer, located from the nasal columella to the philtrum (Fig. 1). After one month of evolution, the ulcer showed involvement of the orbicularis oris muscle, extending to the semi-mucosa of the upper lip (Fig. 2). The patient used cocaine occasionally. Initially, the hypotheses of Wegener's granulomatosis, cutaneous leishmaniasis, and nasal NK/T-cell lymphoma were raised. Complementary tests showed negative p-ANCA and c-ANCA tests. Kidney and liver function tests and blood count were normal. No imaging tests were performed. Histopathology revealed extensive necrosis affecting the epidermis and subcutaneous planes, without signs of vasculitis or malignancy (Fig. 3). After complementary examinations, the diagnosis of this pathology was made. A multidisciplinary approach was carried out with psychiatry and stomatology, and hospital admission was performed, with debridement of the ulcer, in addition to supportive measures and cocaine abstinence, with the patient showing significant clinical improvement of the lesion (Fig. 4).

**Fig. 1:**
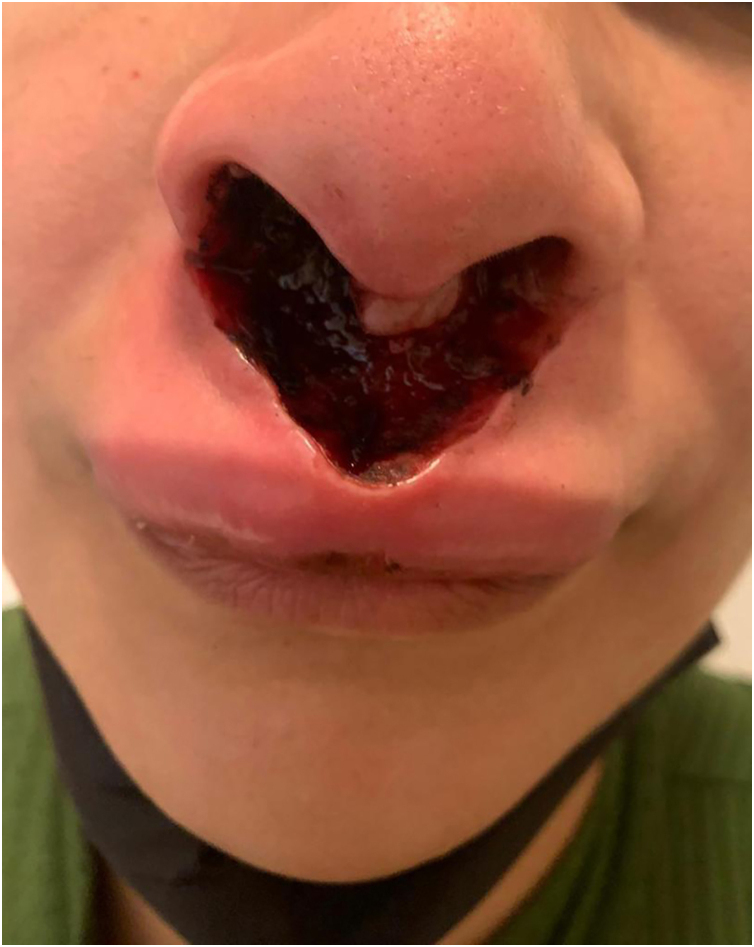
Well-defined necrotic ulcer, located from the nasal columella to the philtrum.

**Fig. 2:**
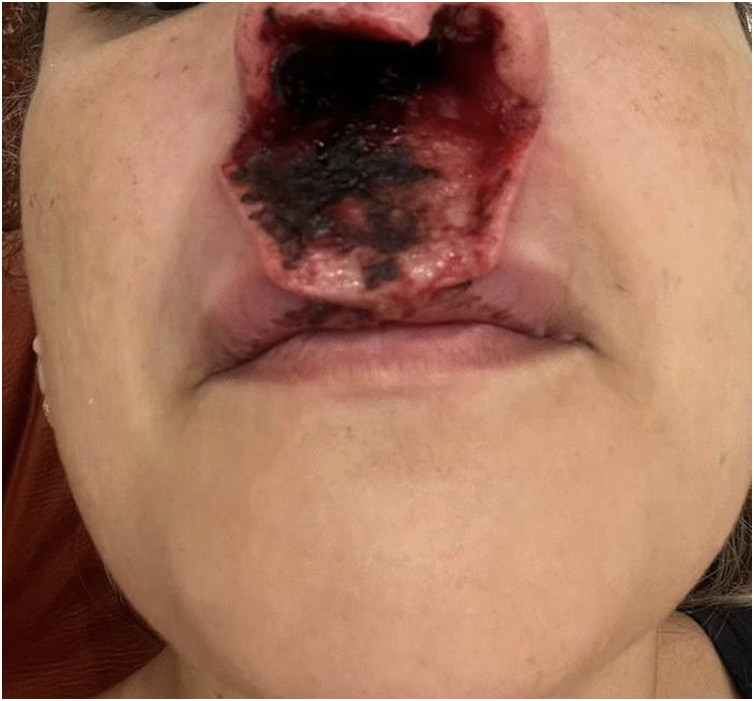
Necrotic ulcer affecting the orbicularis oris muscle and extending to the submucosa of the upper lip.

**Fig. 3:**
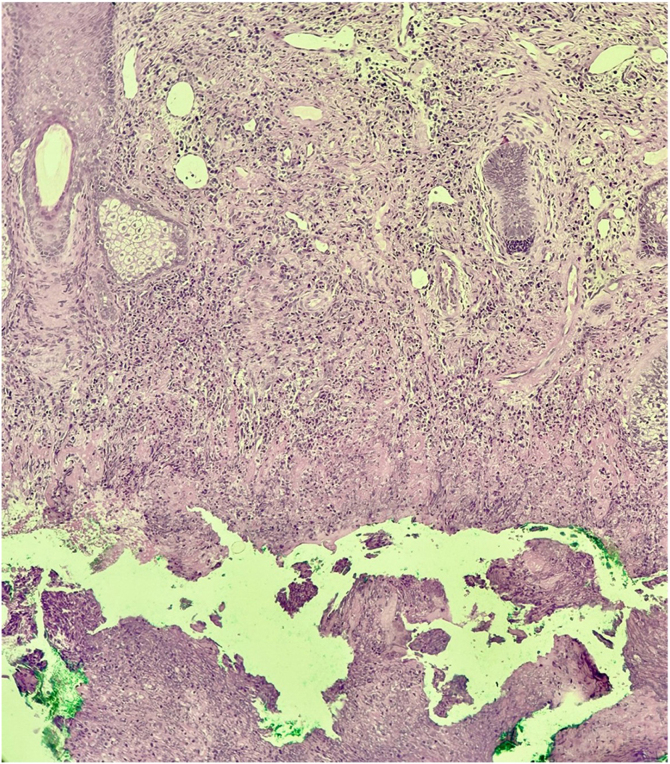
Necrotic area evident in the deep dermis. (Hematoxylin & eosin, ×100).

**Fig. 4:**
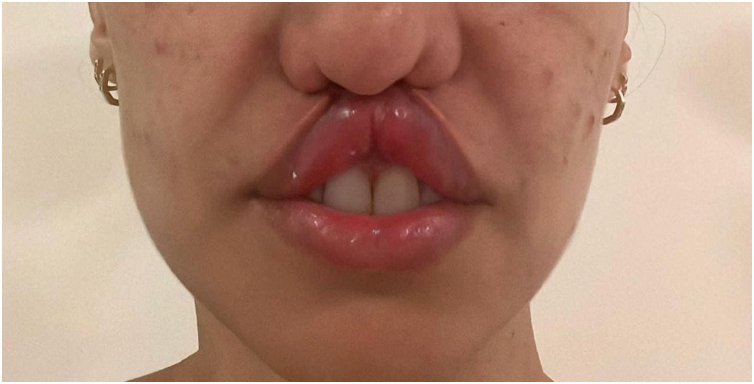
Significant retraction of the upper lip with loss of the philtrum and relevant aesthetic impairment after hospitalization.

Cocaine is a psychoactive drug; its use can become abusive and trigger numerous consequences for the human body through a mechanism of inflammation and nasal necrosis of multifactorial cause, involving a local ischemic vasoconstrictor effect, with one of its complications being the emergence of CIMDL.^1^ Therefore, there is greater susceptibility to structural damage to the mucosa and bone and cartilage structures, with involvement of the nasal septum, hard palate, maxillary bone, and anterior base of the skull.^2^ Consequently, these extensive deformities of the nose and midface can cause chronic damage and loss of structural support of the facial midline.^2^ The prevalence of CIMDL in cocaine users is 4.8%.^3^ However, its cutaneous dissemination is rare. The case described here stands out from the others due to the extensive cutaneous involvement. The literature review showed only one similar case described, reported by Sevinsky in 1995.^4^ The case reported herein exhibited extensive and deep cutaneous involvement, evolving with destruction of the nasal septum.

In 1995, Sevinsky et al. reported a case of a male patient, a chronic cocaine user, who developed CIMDL after intranasal use of the substance.^4^ In the reported case, there was significant cutaneous involvement with total destruction of the nasal septum.

In 2022, Nitro et al. published a systematic review in which they systematized the prevalence of CIMDL involvement sites; 17 studies with a total of 127 patients were eligible. Destruction of the nasal septum occurred in 99.2% of cases. The prevalence of distribution decreased from the lower third of the nasosinusal complex (nasal floor and inferolateral nasal wall, respectively, 59% and 29.9% of patients) to the middle third (middle turbinate and ethmoid; 22.8% of patients) and, finally, to neurocranial structures (7.9% of patients). The authors also proposed a classification based on the distribution patterns of CIMDL. For this purpose, the nasosinusal district was ideally subdivided into four parts that are progressively less involved by cocaine-related lesions, thus allowing grading of the extent of CIMDL. The prevalence of univocal lesions observed is most frequent at the level of the nasal septum (corresponding to Grade 1). The prevalence of invasion progressively decreases in the lower portion of the nasosinusal cavities (palate and/or inferior turbinate, maxillary bone and nasolacrimal duct (Grade 2); therefore, the prevalence reduces even further towards the ethmoid structures (Grade 3) and reaches its lowest prevalence for neurocranial invasion (lamina papyracea, orbit and/or base of the skull; Grade 4).

Given the extensive involvement, it is essential to broaden the understanding of the pathology and to publish reports similar to this case to have a better understanding of this entity, and consequently, an increasingly early diagnosis and, therefore, improve the clinical outcome for those with this condition.

## ORCID ID

Laís Guerra Guedes: 0009-0003-6776-1277

Márcio Martins Lobo Jardim: 0000-0002-8431-3607

Felipe Marinho Rocha de Macedo: 0000-0002-5860-9367

Isabella Cavalcanti Gomes de Sá: 0000-0002-5654-8337

Renata Guerra Galvão Santos: 0000-0002-5201-5066

Daniela Mayumi Takano: 0000-0002-3083-5522

## Financial support

None declared.

## Authors’ contributions

Laís Guerra Guedes: Collection, analysis, and interpretation of data; critical review of the literature; drafting and editing of the manuscript; approval of the final version of the manuscript.

Márcio Martins Lobo Jardim: Design and planning of the study; effective participation in research orientation; approval of the final version of the manuscript.

Felipe Marinho Rocha de Macedo: Conception, analysis, and interpretation of data; approval of the final version of the manuscript.

Isabella Cavalcanti Gomes de Sá: Conception, analysis, and interpretation of data; approval of the final version of the manuscript.

Renata Guerra Galvão Santos: Conception, analysis, and interpretation of data; approval of the final version of the manuscript.

Daniela Mayumi Takano: Conception, analysis, and interpretation of data; approval of the final version of the manuscript.

## Research data availability

Not applicable.

## Conflicts of interest

None declared.
